# Benign Hepatocellular Tumors: A Multidisciplinary Approach

**DOI:** 10.1155/2013/153693

**Published:** 2013-05-28

**Authors:** Paulette Bioulac-Sage, Luigi Grazioli, Türkan Terkivatan, Charissa Chang

**Affiliations:** ^1^Pathology Department, CHU Bordeaux and Inserm U1053, Université Bordeaux 2, 33076 Bordeaux, France; ^2^Department of Radiology, University of Brescia, 25100 Brescia, Italy; ^3^Department of Surgery, Erasmus University Medical Center, 3000 CA Rotterdam, The Netherlands; ^4^Division of Liver Diseases, Mount Sinai School of Medicine, Recanati-Miller Transplant Institute, New York, NY 10029, USA

This special issue is an attempt to cover some new aspects concerning benign hepatocellular tumors: focal nodular hyperplasia (FNH) and hepatocellular adenoma (HCA).

In this special issue, we were fortunate enough to have editors from different countries (France, Italy, The Netherlands, and USA) and from different disciplines (liver pathology, liver radiology, liver surgery, and hepatology) who have contributed 6 papers and 5 contributors from academic centres involved in benign hepatocellular tumors, namely, liver pathologists, radiologists, and molecular biologists from different continents: Europe, North America, and Asia. 

Concerning FNH, if their diagnosis is mainly made by radiologists, some particular cases required a biopsy or even resection. When standard pathological features are atypical, the diagnosis is greatly facilitated by immunohistochemistry, particularly the characteristic pattern of glutamine synthetase (GS), in biopsy as well as surgical specimens, as described by the Bordeaux group.

HCA is the main topic of this issue. This benign rare liver tumor came to medical attention in 1973 when it became clear that oral contraceptives introduced in the USA in 1960 were the main agents responsible for the occurrence of HCA. The first complication identified was bleeding, which was occasionally life threatening, was often the first manifestation of tumor, and often led to surgical resection. The second complication, hepatocellular carcinoma (HCC) transformation, reinforced a surgical approach to treatment in spite of the fact that malignant transformation remains a subject of controversy. 

HCA is a worldwide problem with a clear difference in incidence between Europe and Asia. This difference is mainly explained by the different methods of contraception in the world. In Europe oral contraceptives (OC) are taken by 54, 58, 60, 68, and 75 percent of women in France, Holland, Belgium, Portugal, and Germany, respectively, versus 1% in China, 2% in Japan, and 21% in USA. 

In a study from Japan published in this issue, only 2 of 13 cases of HCA described were found in women taking OC. Presently there is no data concerning the influence of the OC generation on the incidence of HCA. It was thought that the lower levels of estradiol should decrease the risk of HCA. This was not observed. The possible explanation comes from the identification of novel risk factors, namely, obesity and metabolic syndrome.

Exploring the combined experience of US centers, publications reporting HCA in the US were identified through a PubMed search and a review of the literature. Whereas earlier reports of HCA in the US described cases exclusively in women exposed to OC, there is a trend towards an increase in HCAs reported in men, HCAs in the absence of OC use, and increased reports of multiple HCAs. This confirms the experience from European centers and may be a result of newer OC formulations and increasing prevalence of obesity.

Interest in HCA has expanded recently in the first decade of this new century when it was discovered that HCAs are different entities under the control of distinct gene mutations. A molecular classification related to risk factors, pathological features, and risk of transformation in HCC was revealed. As reported in the paper describing molecular classification, three major pathways have been identified which define specific HCA subgroups (1) inactivation of hepatocyte nuclear factor 1A (HNF1A)/transcription factor, (2) activation of the Wnt/b-catenin pathway by *CTNNB1* mutations, or (3) activation of the IL6/STAT3 pathway by somatic mutation of IL6ST, GNAS, or STAT3. The genotype classification led to the phenotypic classification, now well recognized and with specific characteristic immunomarkers. Interestingly, different centres have obtained similar results from the first one published in France. For example in Brussels, as published in this issue, from January 1992 to January 2012, 37 patients underwent surgical resection for HCA. Nine had HNF1*α*-inactivated HCA (H-HCA: 25%) with lack of LFABP expression; 20 had inflammatory HCA (IHCA: 55.5%) showing CRP and/or SAA expression; in 5 patients (14%), *β*-catenin-activated HCA (b-HCA) with GS and nuclear *β*-catenin positivity was diagnosed, two already with HCC. Two cases (5.5%) remained unclassified. One b-HCA exhibited the IHCA histological and immunohistochemical characteristics corresponding to the subgroup of *β*-catenin-activated/inflammatory HCA. Not surprisingly, as suggested in this issue, the percentage of the different subtypes may differ in HCA not linked to OC use such as in glycogenosis, familial adenomatous polyposis, vascular diseases (Budd-Chiari syndrome, portal vein agenesis), drug intake (danazol), in men and young children. Molecular classification is opening a new field of investigation among these examples of nodules which are not presently well characterized. 

 In this issue, it was confirmed that MRI was the method of choice to identify HCA (and differentiate from FNH) and that it was possible to identify the 2 major subtypes (H-HCA and IHCA) in the absence of major remodeling due to bleeding/necrosis. A biopsy can be useful when the diagnosis of HCA and HCA subtypes cannot be made. 

 It was shown that H&E routine histology allows to diagnose >85% of the 2 major HCA subtypes. However, GS is essential to identify b-HCA and b-IHCA. This study demonstrates that a “palliative” diagnostic approach can be proposed, when the panel of specific antibodies is not available. IHCA comprised about 50% of all HCAs and is often associated with obesity as already mentioned. Mild/moderate steatosis is found in the nontumoral liver in a high frequency which is in accordance with the high BMI; of note are the regular findings of sinusoidal dilatation, single arteries, and minute CRP foci which are all features of HCA, as reported by the Groningen's group. These distinct CRP foci are mostly found in cases of multiple IHCA. In academic centers, particularly referral centres for primary liver tumors, immunohistochemistry is essential to understand the natural history of the different HCA subtypes, particularly in women taking OC, and notably with regard to risk for malignant transformation. 

In this issue, the Paris pediatric group studied benign hepatic tumors in children. They are very rare. Most FNH remains sporadic, but predisposing factors exist: long-term cancer survivors, portal deprivation in congenital or surgical portosystemic shunting. HCA is frequently associated with predisposing factors such as glycogenosis type I and III, Fanconi anemia (especially if androgen therapy is administered), congenital portal shunt, and SPSS; of note, the same vascular abnormality can induce 2 different types of nodules. 

Adenomatosis has been mainly reported in germline mutations of *HNF1-A* gene (associated or not with MODY3). Adolescents or adults with MODY3 should be tested for the presence of HCA. Conversely young diabetic patients with H-HCA should be tested for MODY3. 

The Rotterdam group focused its interest on HCA in pregnancy. They come to the important conclusion that pregnancy in women with an HCA up to 5 cm is no longer discouraged in close consultation with the patient, her partner, and members of the liver expert team. In this review you will find also practical guidelines dealing with the diagnosis and management of HCA subtypes in the paper written by authors from different countries. 

We hope that his review will encourage multidisciplinary teams to work in this field. We need to establish guidelines (diagnosis, treatment, and follow-up) in different etiological conditions including yet unknown diseases. As an example, the Japanese team found that in patients with alcoholic cirrhosis, a group of possible inflammatory HCAs was characterized by strong immunoreactivity for serum amyloid A (SAA). They suggested that these SAA-positive hepatocellular neoplasms in alcoholic cirrhosis may be a new entity of HCA, which may have the potential for malignant transformation. They correctly indicate that further studies are needed to clarify genetic changes, monoclonality, and pathogenesis of this new type of hepatocellular neoplasm. Understanding malignant transformation of HCA indeed remains a major objective for the coming years.

*Paulette Bioulac-Sage* *Paulette Bioulac-Sage* *Luigi Grazioli* *Luigi Grazioli* *Türkan Terkivatan* *Türkan Terkivatan* *Charissa Chang* *Charissa Chang*

